# Heavy Metals Accumulation in Tissues of Wild and Farmed Barramundi from the Northern Bay of Bengal Coast, and Its Estimated Human Health Risks

**DOI:** 10.3390/toxics10080410

**Published:** 2022-07-22

**Authors:** Tanha Tahity, Md. Rakeb Ul Islam, Nurer Zaman Bhuiyan, Tasrina Rabia Choudhury, Jimmy Yu, Md. Abu Noman, Mohammad Mozammal Hosen, Shamshad B. Quraishi, Bilal Ahamad Paray, Takaomi Arai, Mohammad Belal Hossain

**Affiliations:** 1 Department of Fisheries and Marine Science, Noakhali Science and Technology University, Noakhali 3814, Bangladesh; tanha.tahityn@gmail.com (T.T.); mrakeb_82bau@yahoo.com (M.R.U.I.); 2 Independent Researcher, Mustankivenkatu 2C 38, 00980 Helsinki, Finland; zaman2050@gmail.com; 3 Analytical Chemistry Laboratory, Chemistry Division, Atomic Energy Centre Dhaka (AECD), Bangladesh Atomic Energy Commission, Dhaka 1000, Bangladesh; mmhosen@baec.gov.bd (M.M.H.); mumu3222@baec.gov.bd (S.B.Q.); 4 School of Engineering and Built Environment, Griffith University, Brisbane, QLD 4111, Australia; jimmy.yu@griffith.edu.au; 5 State Key Laboratory of Biogeology and Environmental Geology, China University of Geosciences (Wuhan), Wuhan 430074, China; abu.noman.nstu@gmail.com; 6 Department of Zoology, College of Science, King Saud University, P.O. Box 2455, Riyadh 11451, Saudi Arabia; bparay@ksu.edu.sa; 7 Environmental and Life Sciences Programme, Faculty of Science, Universiti Brunei Darussalam, Jalan Tungku Link, Gadong BE1410, Brunei; takaomi.arai@ubd.edu.bn

**Keywords:** heavy metals, Barramundi fish, farmed fish, wild fish, health risks, Bangladesh

## Abstract

Globally, both natural water bodies and aquaculture systems are being severely contaminated by heavy metals due to rising anthropogenic activities. Fish living in aquatic environments can easily accumulate metals in their bodies, which can then be transferred to consumers and put them at risk. In this study, metal concentrations (Pb, Cd, Cr, As, Mn, Cu, Zn) in different organs (gill, liver, and muscle) of farmed and wild Barramundi (*Lates calcarifer*) fish from the northern Bay of Bengal were evaluated to quantify and compare contamination levels and related human health risk. Heavy metal concentrations were higher in liver tissues of farmed Barramundi than in wild Barramundi, with the following relative mean values in the liver, gills, and muscle: Zn > Cu > Pb > Mn > Cd > Cr > As; Zn > Cr > Cu > Pb > Mn > Cd > As; Zn > Pb > Cu > Cr > Mn > Cd > As; Zn > Pb > Cu > Cr > Mn > Cd > As; and Zn > Pb > Cu > Cr > Mn > Cd > As, respectively. The differences in heavy metal accumulation observed between farmed and wild fish were probably related to the differences in their environmental conditions and dietary element concentrations. However, ANOVA indicated that the variation of metals in wild and Barramundi was not statically significant. Pb concentrations in the liver tissue of farmed Barramundi exceeded the national and international threshold limits, whereas concentrations of other metals were within the limit. Among the examined organs in both fish species (wild and farmed), muscle had the lowest concentration compared to others, and liver was the target organ for Pb, Cu, and Cd accumulations. Metals such as Zn and Mn exhibited higher concentration in the gills. However, all the studied heavy metals were below the maximum permissible limits of national and international standards, but the mean concentrations of Pb and Cd values in the liver of farmed Barramundi exceeded all international and national guidelines. Based on the contamination factors (CF) and pollution indices (PLI and MPI), the degree of contamination in the fish organs was as follows: gills > liver > muscle. The major accumulation tissues for both farmed and wild fish were found to be the gills (MPI = 0.970) and the liver (MPI = 0.692). Based on the estimated daily intake (EDI), the fish samples examined in this study are safe for human consumption as within the recommended daily allowance (RDA) range established by various authorities. According to the Target Hazard Quotient (THQ) and Carcinogenic Risk (CR) calculations, though the Barramundi fishes depicted no potential hazard to humans, farmed fish posed a higher health risk than wild fish.

## 1. Introduction

Humans rely heavily on fish for their nutritional needs since fish have a higher trophic position i.e., tertiary level in the food chain. Fish are generally high in essential minerals, vitamins, and unsaturated fatty acids, in addition to being a good source of protein [1]. As a result, to achieve daily omega-3 fatty acid requirements, the American Heart Association recommends eating fish at least twice a week [2]. However, with rapid industrial expansion, fish habitats are today under serious threat of pollution. In developing countries such as Bangladesh, most industries release their solid and liquid wastes or pollutants without any treatments. Among the pollutants, heavy metals have become a major concern worldwide due to their toxicity, inherent persistence, non-biodegradability, and accumulative tendencies [3,4]. Fish have the ability to accumulate heavy metals in their tissues at higher levels than environmental quantities due to absorption along the gill surface, kidney, liver, and gastrointestinal tract wall [5]. Some toxic heavy metals are not metabolized by the fish body and accumulate in the soft tissues, and hence they become magnified through the biomagnification process. If the accumulation in fish tissues exceeds the maximum permitted concentrations, fish consumption can cause a human health risk as some metals, such as Hg, Cr, Cd and As, can damage the kidneys, liver and nervous system [1]. As a result, the increased concentration of heavy metals in fish has a significant impact on the human body [6]. Hence, fish are considered one of the most common bio-indicators of contaminants to explore anthropogenic effects on the environment and human health [7].

Heavy metals enter the aquatic food chain through two routes: (1) direct water and food consumption through the digestive tract, and (2) non-dietary pathways such as muscle and gills [8]. Industrial effluents, municipal sewage, agrochemical waste products, geological weathering, and atmospheric deposition are all regarded as major sources of heavy metals in the aquatic environment around the world [9]. These high inputs of heavy metals in the aquatic ecosystems make them able to bio-accumulate in aquatic species including fish. Heavy metal accumulation by organisms, on the other hand, can be passive or selective, and variations in heavy metal accumulation by organisms could be due to variances in assimilation, egestion, or both [5]. Heavy metals and metalloids are serious pollutants for all living species, including humans, at increasing concentrations. Excessive quantities of Hg, As, Pb, and Cd components, for example, are harmful to living cells and can cause illness or death if inhaled for long periods of time [10].

Heavy metals have detrimental impacts on aquatic living organisms and their ecosystems. Once released into the aquatic ecosystems, they persist in the environment for a long time for their non-biodegradability. In the aquatic environment, metals are dispersed throughout the water column, deposited in sediments, and eaten by biota. Due to metal desorption and remobilization processes, the sediments act as a long-term source of contamination for the dwelling species and food chain [5]. As a result, they can deteriorate water quality (e.g., reduce the dissolved oxygen levels and increase the acidity), modify sediment geochemistry (through accumulation, binding fine particles, co-precipitation with Fe or Mn oxides and leaching) and cause extensive damage to physiological activities (e.g., growth, respiration and reproduction) of the organisms or even mass mortality [6]. In the long run, the cumulative and irreversible accumulation of heavy metals in various organs of aquatic species causes a variety of diseases, putting the aquatic biota and other organisms at risk. Aquaculture species are exposed to these heavy metals through food consumption, water uptake by gills, inedible particle eating, and dermal absorption. Fish, as one of the most important aquaculture species in the food chain, can accumulate significant levels of certain metals. They can accumulate heavy metals from their environment and can absorb them through their food as well. Unlike organic molecules, the majority of metals cannot be digested or converted into less harmful compounds, causing them to absorb in the body and transfer from one trophic level to another, potentially causing health concerns [6]. Therefore, it is essential to improve our understanding on toxic metal levels in commercially important aquaculture species.

The monitoring of metal levels in fish and other foods is crucial since they not only offer nutritional benefits but also occasionally put human health at risk. Numerous techniques and tools have been devised and put to use in recent years for reliable and precise measurement of metals [5]. The traditional technique for detecting metals is atomic absorption spectrometry (AAS). Later, other efficient techniques were developed, such as inductively coupled plasma-optical emission spectrometry (ICP-OES), portable atmospheric pressure discharge plasma (APDP), flame atomic absorption spectrophotometer (FAAS), atomic fluorescence spectrometry (AFS), and laser-induced breakdown spectrometry (LIBS). AFS has the benefits of both AES and AAS while addressing their respective drawbacks. An ideal and widely used analytical technique for a lab setting is ICP-AES. LIBS is ideal for real-time and online detection of environmental heavy metal contaminants. However, professionals and academics have begun to pay attention to portable, low-cost air pressure discharge plasma (APDP) technology, which has emerged as the best way to test for trace elements. Compared to conventional detection methods, APDP has the benefits of simple equipment and a low price.

Previous research has indicated that direct discharge of urban waste, untreated industrial effluents, and agrochemicals into Bangladesh’s rivers and tributaries has had a serious impact on aquatic ecosystems [11]. Furthermore, sewage water from livestock farms, agriculture, and even chemical product industries has recently infiltrated aquaculture via polluted river water. Agricultural non-point source pollution has become worse, and fishermen’s management has been unscientific [12]. Moreover, due to inexpensive investment and simple regular management requirements, Bangladesh’s farmed fish output has increased tremendously in recent years [13]. The presence of heavy metals in the environment and feed at aquaculture sites might result in heavy metal accumulation in aquaculture food items. Therefore, in addition to wild fish species, metal pollution in cultivated fish is a matter of concern for human health. The Barramundi (*Lates calcarifer*) is a popular commercial fish in Asia, particularly on the Indian subcontinent, including Bangladesh. They inhabit a variety of aquatic ecosystems including freshwater, brackish and marine environments, such as streams, wetlands, estuaries and coastal waters, and feeds on fish, crustaceans, molluscs and other invertebrates. Barramundi fish is being cultured in coastal water throughout Bangladesh. Therefore, the Barramundi is an ideal species for studying metal bioaccumulation in the wild and in the cultured area, as well as the consequences for human health.

Much research has been carried out to evaluate the levels of heavy metals in fish and the risk of human exposure [3,5,7,12,13]. Metals have been reported to be deposited in a variety of organs, including the bone, brain, digestive system, gonads, heart, kidneys, and liver, according to a number of studies [14,15,16]. However, the majority of the studies have focused on wild fish [17,18,19,20], and the comparative metal accumulation in cultured and wild fish are scant [21,22,23], and particularly it has yet to be studied in Bangladesh. There was no detailed study found on metal levels in different organs of Barramundi. Therefore, this study was conducted to (i) determine the heavy metal concentration in three parts (liver, gill, muscle) of cultured and wild Barramundi fish, and (ii) to compare and estimate the heavy metal contamination, bioaccumulation, and potential health risk to humans through its consumption.

## 2. Materials and Methods

### 2.1. Study Area

**Figure 1:**
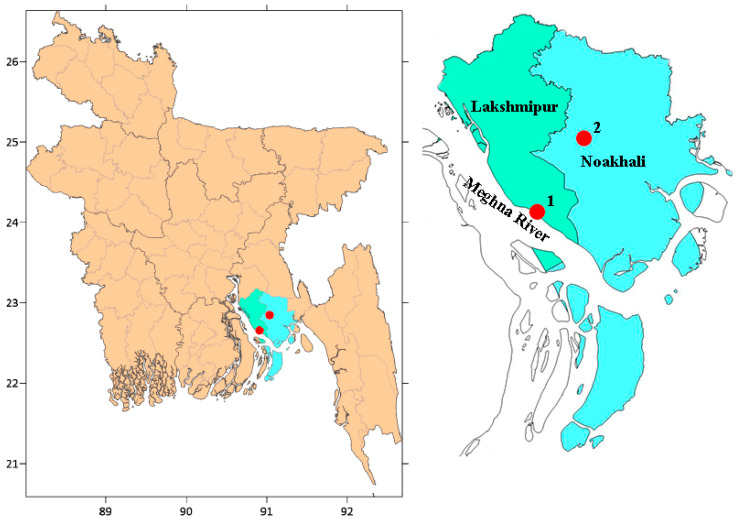
Sampling location of the Wild (site 1) and Cultured (site 2) Barramundi fish in Bangladesh.

The Meghna River, which comes into Bangladesh from India’s hilly eastern regions and originates in the Barak River, passes through the Kishoreganj district. The river, however, flows into the Bay of Bengal, and the well-known Meghna River Estuary is located in the southern region of Bangladesh. Wild Barramundi were collected from the Char Alekjander region (22°40′0″ N, 90°54′0″ E), which is in the heart of the Meghna River. Char Alexander is a freshwater beach near the market of the Alexander Municipality, 40 km from the Noakhali district (Figure 1). The farmed Barramundi samples were collected from the fish farms Suborno Agro (22°31′13.904″ N, 91°8′7.087″ E) and Hatiya, Noakhali. Fish farms mainly depend on artificial feeds commercially available in the market. The first site (from where the wild Barramundi were collected) was located near the industrial area. The primary sources of pollution in the study areas are domestic and industrial effluent, runoff and pollution from road transport. Therefore, the study area provides a strategic position for studying the industrial and commercial hub. Furthermore, the anthropogenic activities have resulted in the release of a variety of pollutants into the river and culture areas of the Noakhali district.

### 2.2. Sample Collection and Preservation

To compare the metal concentrations in different organs of Barramundi, samples of wild Barramundi (*Lates calcarifer*) fish were collected with the help of local fishermen from the lower Meghna River area (from Alekjander Upazilla of Laxmipurdistrict), and farmed Barramundi was purchased from different local farms of Subarnachar, Charbata and Hatiya. In total, 60 samples (30 samples for each condition; 6 samples from five specimens) from both wild and farmed Barramundi were analyzed for metal levels in gills, liver and muscle. The fish farmers agreed to participate in the study on the condition that their identities not be published, hence the sampling farms are anonymous in this report. The total length and weight of each captured fish were measured to the nearest centimeters and grams before dissection. The fish lengths were between 22 to 29 cm, and the wet weight ranged from 314 to 348 g (Table S1). The length and weight of the collected wild and farmed specimens did not significantly vary at the 5% level (W = 15, *p* = 0.06 ). Each sample was packed in zip-lock polythene bags and transported to the Analytical Chemistry Laboratory, Atomic Energy Centre, Dhaka, Bangladesh, to avoid contamination. The samples were carefully cleaned with deionized water after being delivered to the laboratory. Then, using a clean stainless-steel knife, the eatable parts (muscle) of each sample were separated and sliced into little pieces. The samples were then homogenized using a blender and about 200 g of test portions was stored at −20 °C.

### 2.3. Sample Preparation, Analysis and Quality Control

Approximately 2 g of fish tissues (liver, gill, muscle) was weighed from each sample, and two replicate samples were taken from each sample group (Wild and Culture). The digestion of the samples occurred in a closed-vessel microwave digester (MARS-5; CEM Corporations, Charlotte, NC, USA) with 0.5 g sample, 6 mL ultra-pure concentrated nitric acid and hydrogen peroxide (1 mL). The heating arrangements in the microwave digester consisted of three steps: 15 min of ramping at 180 °C, 10 min of holding at 180 °C, and at the end 15 min of cooling. The samples were then diluted in deionized water (resistivity > 18 MΩcm, manufactured using an E-pure system) to a final volume of 10 mL (Thermo Scientific, Waltham, MA, USA). Furthermore, for the determination of As, digested samples were pretreated with ascorbic acid (Merck, Darmstadt, Germany) and potassium iodide (Merck, Darmstadt, Germany) prior to analysis.

An Atomic Absorption Spectrophotometer (Model no. Varian, DuoAA240FS and AA80Z) with Zeeman background correction system fitted with graphite furnace (GTA 120) and an auto sampler was used to determine the amounts of Cd, Cr, and Pb (PSD 120). For As, a hydride generator (HG-AAS) approach using a hydride vapor generator was used (VGA 77). The Flame-AAS (Varian AA240FS) was used to quantify the concentrations of Mn, Cu, and Zn. Acetylene and argon gases have purity levels of 99.999 and 99.99%, respectively. For Pb (283.3 nm and slit 0.5 nm), Cd (228.8 nm and slit 0.5 nm), Cr (357.9 nm and slit 0.2 nm), As (193.7 nm and slit 0.5 nm), and Hg (253.7 nm and slit 0.5 nm), hollow cathode lamps were employed, and they were operated in accordance with the manufacturer’s recommendations. Atomic signals for Pb, Cd, and Cr in peak area mood as well as As and Hg in integration mood were measured.

The Analytical Chemistry Laboratory of Atomic Energy Centre, Dhaka, Bangladesh is an ISO/IEC 17025 accredited laboratory and for ensuring quality of the tests, the laboratory has been routinely continuing both internal and external quality control programs in accordance with guidelines. For internal QC, the laboratory maintains several steps such as measurement of blank samples, control standard samples, spike check/recovery check, replicate analysis, the maneuver of certified reference materials, and use of control charts to monitor the accuracy of the data regularly. As a part of the external quality control, the laboratory also participates in a number of proficiency tests per year provided by international proficiency testing providers and securing the required satisfactory scores. Moreover, for the recovery of the analytical procedure employed for the analysis of heavy metals in the present study, a certified reference material Dorm -2 produced at the National Research Council of Canada (NRC-CNRC) was measured. The mean recoveries were from 91 to 99%, indicating the suitability of the method.

### 2.4. Statistical Analysis

Parametric one-way ANOVA (analyses of variance), Welch’s ANOVA and Wilcoxon test were conducted to evaluate differences in element concentrations between gills, muscle and liver in farmed and wild fish. The levels were considered significantly different at *p* < 0.05. Spearman rank correlations were used to identify the relationships of metals in gills, liver and muscle. The metal concentration data were log-transformed before further multivariate analysis. Pearson’s correlation analysis (CM) and principal component analysis (PCA) were performed to analyze the origin and associations of metals. Mathematical calculations, CM and PCA were carried out using Microsoft Excel (version 10; Microsoft, Washington DC, USA) and PAST (version 3; Palaeontological Association, Oslo, Norway). Graphical representation of the metal concentration was plotted with GraphPad Prism (version 7; GraphPad, San Diego, CA, USA).

### 2.5. Assessment of Contamination Level

Several indices have been developed and frequently used for metal contamination level assessments in sediment, water and aquatic organisms [24,25,26,27]. In this study, some commonly used indices e.g., contamination factor (CF), pollution load index (PLI) and metal pollution index (MPI) were used.

#### 2.5.1. Contamination Factor (CF)

The contamination factor (CF) for metals was derived using metal concentrations in fish [24]:

CF = C_metal_/C_background_ (1)

where C_metal_ is the metal concentration in fish, and C_background_ is the background concentration of the metal. In this investigation, the lowest metal concentration was used as a baseline or background value. Low contamination is denoted by a CF value of less than 1, moderate contamination is denoted by a CF value of less than 3, major contamination is denoted by a CF value of less than 6, and extremely high contamination is denoted by a CF value of greater than 6 [24].

#### 2.5.2. Pollution Load Index (PLI)

The pollution load index (PLI) was calculated using the concentrations of seven heavy metals to measure the organism’s quality [6,25]:

PLI = (CF_1_ × CF_2_ × CF_3_ ⋯⋯ × CF_n_)^1/n^ (2)

where n is the number of metals examined and CF_1_ is the contamination factor of first concerning metals, CF_2_ is the contamination factor of second concerning metals, CF_3_ is the contamination factor of third concerning metals, and CF_n_ is the contamination factor of nth metals. When the PLI value is less than one, the degree of pollution is low, however any number greater than one indicates that the site and estuary’s quality is deteriorating [25].

#### 2.5.3. Metal Pollution Index (MPI)

The metal pollution index (MPI) is an integrated approach to assess heavy metal pollution. The MPI was estimated using following equation [26,27]:

MPI = (M_1_ × M_2_ × M_3_ ×⋯⋯ × M_n_)^1/n^ (3)

where n is the number of examined metal, M_1_ is the concentration of first metal, M_2_ is the concentration of second metal, M_3_ is the concentration of third metal and M_n_ is the concentration of nth metal (mg/kg dry wt) in the muscles of fish.

### 2.6. Human Health Risk Assessment

#### 2.6.1. Estimation of Daily Intake (EDI)

The consumption of metals found in meals is used to assess human health concerns [28]. To do so, the EDI must be determined, which is based on the metal concentration in foods as well as daily consumption of certain food items [29]. The following equation was used to determine the EDI, provided by the United States Environmental Protection Agency [USEPA] [30,31]

$$\mathrm{EDI}=\frac{\mathrm{CN}\times \mathrm{IGr}}{\mathrm{BWi}}$$

where CN is the selected metal concentration (mg/kg wet weight basis), IGr is the ingestion rate. Adults’ IGr is 55.5 g/day, whereas children’s IGr is 52.5 g/day [28,29]. BWi is the body weight of the consumers (adults—70 kg and children—15 kg) [32].

#### 2.6.2. Targeted Hazard Quotient (THQ) for Non-Carcinogenic Risk

THQ was assessed in order to estimate the level of risk associated with pollutant exposure [33]. The ratio of estimated daily intake (EDI) to oral reference dose (RfD) is used to compute THQ. Metal RfD (mg/person/day) is 0.0003, 0.002, 0.001, 0.003, and 0.3 for As, Pb, Cd, Cr, and Cu, respectively [32]. There are no non-carcinogenic risk effects when the ratio is less than unit limit, and the equation is as follows [28,34]:

$$\mathrm{THQ}=\frac{\mathrm{ED}\;\times \;\mathrm{Ep}\;\times \;\mathrm{EDI}}{\mathrm{RfD}\;\times \;\mathrm{AT}}\times 10^{-3}$$

where ED denotes the period of exposure (65 years) [32]; E_P_ stands for annual exposure frequency. For each metal content, the EDI is the anticipated daily consumption. Non-carcinogens (ED, Ep) have an average time of AT.

#### 2.6.3. Hazard Index (HI)

Through food consumption, humans are exposed to a variety of pollutants [35]. To determine the additive impacts of those pollutants, the following equation is used to calculate HI for various pollutants [36]:

$$\mathrm{HI}=\sum_{i=k}^{n}THQ$$

THQ stands for the risk values of numerous elements extracted from fish samples [37]. Consumers will incur significant non-carcinogenic health risk if the HI value is more than unit limit [37].

#### 2.6.4. Carcinogenic Risk (CR)

CR is the cumulative risk of cancer in an individual over a lifetime as a result of exposure to a serious carcinogen [37]. The CR is determined by multiplying the carcinogenic slope factor of the metal contents following the equation below [38]:

$$\mathrm{CR}=\frac{\mathrm{ED}\;\times \;\mathrm{EP}\times \;\mathrm{EDI}\;\times \;\mathrm{CSF}}{\mathrm{AT}}\times 10^{-3}$$

where CSF stands for oral slope factor of carcinogens (mg/kg/day) [31].

## 3. Results and Discussion

### 3.1. Concentrations of Metals in Cultured & Wild Barramundi

**Figure 2:**
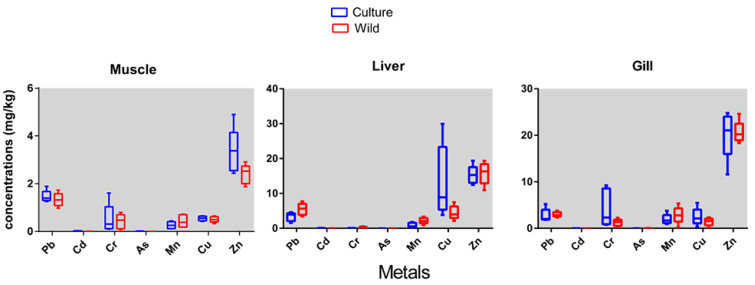
Concentration of metals in muscle, liver and gill tissue of cultured and wild Barramundi fish.

Concentrations of heavy metals (Pb, Cd, Cr, As, Mn, Cu, Zn) in the muscles, liver and gills of cultured and wild Barramundi fish are presented in Table 1. The relative mean concentrations in the liver, gills and muscle of cultured Barramundi fish were as follows: Zn > Cu > Pb > Mn > Cd > Cr > As; Zn > Cr > Cu > Pb > Mn > Cd > As; and Zn > Pb > Cu > Cr > Mn > Cd > As, respectively. The relative mean concentrations in the liver, gills and muscle of wild Barramundi fish were as follows: Zn > Pb > Cu > Mn > Cr > As > Cd; Zn > Mn > Pb > Cu > Cr > As > Cd; and Zn > Pb > Cu > Mn > Cd > As > Cr, respectively (Figure 2).

One-way analyses of variance (ANOVA) confirmed that the overall metal concentrations between wild and farmed Barramundi fish varied significantly (ANOVA: F = 9.316, *p* < 0.05). This might be due to the differences in metal concentrations in the water column and sediments between the aquaculture and wild environment. Higher metal concentrations were also found at the culture site of Carp, *C. carpio* in a previous study [21] because of the release of metals from uneaten fish food and fish excreta. The key food sources for cultured fish are artificial feed and small trash fish. Owing to a lack of uptake from the dissolved phase (i.e., water and sediment), heavy metals from food sources are the major contributor of heavy metal bioaccumulation in cultured fish [39].

However, when compared to the metal concentration in individual organs of wild and farmed Barramundi, the results were different from the overall concentrations. ANOVA confirmed that metal concentrations in liver of wild and farmed Barramundi did not vary significantly (F = 21.92, *p* < 0.05). Nevertheless, Wilcoxon tests showed that the individual metal (Pb, Mn, Cu and As) concentrations in liver varied significantly between wild and farmed sea bass (for Pb: W = 15, *p* = 0.04, for Mn: W = 15, *p*= 0.04, for Cu: W = 15, *p* = 0.04, for As W = 15, *p* = 0.04), and did not vary for Cd (W = 14, *p* = 0.07), Cr (W = 12, *p* = 0.22), or Zn (W = 15, *p* = 0.89). For gills, ANOVA demonstrated that metal concentrations in wild and farmed Barramundi did not vary significantly (F = 123.8, *p* < 0.05) either. Nevertheless, in respect of individual metals, Wilcoxon tests showed that the concentrations in gills did not differ significantly between wild and farmed sea bass (for Pb: W = 11, *p* = 0.35, for Mn: W = 12, *p* = 0.22, for Cu: W = 11, *p* = 0.34, for As W = 8, *p* = 0.28, for Cd: W = 14, *p* = 0.08, for Zn: W = 8, *p* = 0.89), except for Cr (W = 15, *p* = 0.04). For muscles of wild and farmed sea bass, ANOVA indicated the metal concentrations were not significantly varied (F = 1.977, *p* = 0.08). However, when we compared the individual metal levels in muscles of wild and farmed Barramundi, As (W = 15, *p* = 0.04) and Mn (W = 15, *p* = 0.04) levels varied, and other metals did not vary (for Cd: W = 14, *p* = 0.08, for Cr: W = 9, *p* = 0.69, for Cu: W = 11, *p* = 0.34, fo Zn, W = 12, *p* = 0.22).

Overall, the heavy metal concentrations in the cultured and wild Barramundi fish indicated that the liver possessed the highest amounts (e.g., Pb: 5.5 ± 1.6 in liver; 3.00 ± 0.6 in gills and 1.33 ± 0.30 in muscle) of all examined metals except Zn, followed by the gills and muscle (Figure 2). The essential metals Zn and Cu and nonessential metal Pb indicated higher bioaccumulation in the liver, whereas the highest levels of Cr, Mn, and Zn were in the gills. Among the metals, concentrations of Cd and As were almost similar in the organs of both the cultured and wild species (Figure 2). The accumulation of essential metals in the liver is likely linked to its role in metabolism [40]. High levels of Zn and Cu in hepatic tissues are usually related to natural binding proteins such as metallothioneins [41] which act as an essential metal store (i.e., Zn and Cu) to fulfill enzymatic and other metabolic demands [42,43]. Similar results of high Zn, Cu and Cd in the liver were observed in many field studies [40,44]. However, the Barramundi fish also tend to accumulate Zn, Cr and to some extent Mn in gills. Generally, gills are the main route of metal ion exchange from water [45] as they have very large surface areas that facilitate rapid diffusion of toxic metals [46]. Therefore, it is suggested that metals accumulated in gills might be concentrated from water. This is in agreement with the findings of Moore et al. [47]. However, in this study, the metal concentration of water was not analyzed, hence a direct relationship between metals in gills and water could not be placed. However, in general, the concentration of metals in the gills reflects the level of the metals in the waters where the fish live, whereas the concentration in liver and kidney represents the storage of metals [48].

Findings of our study revealed that the mean Pb concentration in both cultured and wild species was highest in the liver. However, the wild species (5.5 mg/kg) accumulated higher Pb than cultured species (3.4 mg/kg) (Table 1). Both of these values exceed all international and national guidelines (Table 2). Pb is a non-essential element that has been linked to neurotoxicity, nephrotoxicity, and a variety of other health problems [49]. However, the concentration of Pb was in line with the results of Aegean Sea [22], where the range of Pb in cultured and wild Barramundi was 1.03 mg/kg and 0.84 mg/kg, respectively. The concentrations of our study in muscle tissues of cultured and wild species were almost similar to cultured and wild Rainbow trout [50], where the mean concentrations of Pb in muscle tissues were reported as 1.108 mg/kg for cultured species, and 1.201 mg/kg for wild species (Table 3). Rashed et al. [50] found that elevated Pb levels in fishes obtained from freshwater ecosystems were affected by extended agriculture, poultry farms, textile, industrial and other activities.

The mean value of Cd in the liver of cultured (0.1 mg/kg) species was higher than the wild species (0.02 mg/kg) (Table 1). Cd in liver of cultured species was higher than the FAO-recommended limit [51] (Table 2). Other organs such as the gills and muscles possessed the concentration values of 0.034 mg/kg and 0.022 mg/kg, respectively, but did not exceed the WHO [52] and MAFF [53] permissible limits (Table 4). However, the concentration level in the cultured species was higher than the previously reported concentrations in cultured fish species of Bangladesh [54] and cultured carp fish in Japan [21]. In the wild species, metal concentrations in all the organs were within the limit of international and national guidelines (Table 2). However, the results of the muscle and liver tissues were far below the findings in cultured and wild Rainbow trout in Iran [50] and cultured and wild carp [21] (Table 3). In general, manufacturing processes (such as smelting or electroplating) and chemical fertilizers are reasons for the increase in Cd in the aquatic environment.

The lowest Cr levels in Barramundi gills were 1.228 mg/kg in wild Barramundi, whereas the highest was 4.257 mg/kg in cultured Barramundi. The concentration of Cr in cultured Barramundi in this study was consistent with Fallah et al. [21], where they reported Cr concentration of 0.57 mg/kg in cultured rainbow trout. The mean Cr concentrations of 1.274 mg/kg were recorded in cultured fish species in a previous study of Bangladesh [54], which is higher than our findings. However, some other studies from different parts of the world reported that Cr was lower than our findings [21,22,23]. Moreover, the muscle and liver tissues of wild Barramundi of our study had higher mean Cr concentrations than the earlier reported wild carp [21] and lower than cultured and wild Rainbow trout [50]. However, the presence of Cr in river fish samples could be attributable to waste water from various eastern region industries such as dyeing and tanning, textile, photography, paints and inks, and river runoff from upstream agricultural fields [55].

The concentration of As in cultured and wild Barramundi ranged from 0.002 to 0.032 mg/kg in the liver, gills, and muscle. The highest value of 0.032 mg/kg was found in the gills of wild species, which is higher than the maximum permissible concentration of the metal in fish species set by WHO [56], but lower than the limit of FAO and MOFL [51,57] (Table 2). Arsenic has been classified as a human carcinogen. Long-term exposure to arsenic has been linked to skin, vascular, nervous system, and cancer issues [58]. However, the As level in cultured Barramundi was lower than that of cultured fish species in Bangladesh [54], cultured and wild carp of muscle and liver tissue in Japan [21], and rainbow trout in Iran [50], but higher than that of other cultured fish from Honghu Lake, China [23].

In the present study, the concentration of Mn was highest in wild Barramundi compared to the culture Barramundi (Figure 2). Mn concentrations in wild and cultured species were highest in the gills (2.854 mg/kg and 1.939 mg/kg) compared to other organs (Table 1). In cultured species, the metals in the gills exceeded the maximum permissible limit (Table 2). The Mn concentrations in the gills and liver were higher than the WHO [56] and FEPA [59] allowable limits, but the muscle value was acceptable (Table 3). Manganese (Mn) is an essential element with a wide range of biological applications. However, ingesting large amounts of this element can cause neurologic and psychiatric problems [60]. The cultures and wild Barramundi muscle and liver values were lower than previous studies of cultured and wild Barramundi from Iran (6.262 mg/kg, 18.871 mg/kg; 13.932 mg/kg, 31.794 mg/kg [49]). However, the Mn in the cultured and wild Barramundi was higher than cultured and wild carp (0.1766 to 0.390 mg/kg) [21].

Among the cultured and wild Barramundi, the concentration of Cu was highest in cultured Barramundi. In cultured species, the observed mean concentration was given in following order: liver (13.233 mg/kg) > gills (2.464 mg/kg) > muscle (0.543 mg/kg). The concentrations of Cu in all organs of cultured Barramundi were lower than the permissible limit of WHO [52], FAO [51], and MAFF [53] (Table 2). In wild species, concentration levels of Cu were also lower than all international guidelines except the European Union [61]. Although optimum copper is necessary for health, especially in the synthesis of hemoglobin and several vital enzymes, excess amounts of it can harm the liver and kidneys [34,38]. In the previous literature, Cu levels in cultured and wild species were reported in the range of 3.87–2.96 mg/kg from Aegean Sea [22]; muscle and liver values 21.813–58.494 mg/kg from Iran [50]; 0.85–0.42 mg/kg from Honghu Lake, China [23]. In this study, wild species’ muscle and liver values were higher than Kasumigaura, Japan (0.249 mg/kg, 0.741 mg/kg) [21] (Table 3).

The mean Zn concentrations in different organs of wild Barramundi were in the following order: gills (20.632 mg/kg) > liver (15.804 mg/kg) > muscle (2.401 mg/kg) (Table 1). In cultured Barramundi, the mean concentration of Zn in different organs was in the following descending order: gills (20.216 mg/kg) > liver (15.284 mg/kg) > muscle (3.532 mg/kg) (Table 1). The gills of the wild species had the highest Zn concentration (20.632 mg/kg) among all organs analyzed in the cultured and wild Barramundi. This finding revealed that the Zn concentration might be higher in the wild environment near the sampling area. However, the Zn concentration in all the organs in both cultured and wild Barramundi were within all international and national standards (Table 2). Zn contents in the literature have been reported in the range of 18.94–16.30 mg/kg dry weight in cultured and wild species from Honghu Lake, China [23]; muscle and liver values of cultured and wild species were 20.973, 81.068 mg/kg and 46.742, 125.250 mg/kg mg/kg, respectively from Iran [50], and 45.1–43.6 mg/kg in fish species from Aegean Sea [22] (Table 3). All those values were higher than the concentration of Zn recorded in the present study.

### 3.2. Environmental Risk Assessment

The contamination factor (CF), pollution load index (PLI), and metal pollution index (MPI) can be used to assess the degree of heavy metal contamination in organisms [27]. The PLI is a function of contribution of the studied metals and offers an assessment of the sample’s overall toxicity status (Pb, Cd, Cr, As, Mn, Cu, Zn). The obtained PLI values indicated that the quality of the investigated organisms, both cultured and wild Barramundi, is not declining. The estimated value for CF in fish samples showed a range of 0.001–0.294, indicating a low contamination region for cultured Barramundi. Furthermore, wild Barramundi were in a low pollution zone with a contamination range of 0.0002–0.25 (Table 4). MPI was previously used to assess metal toxicity in various aquatic organisms, and to compare levels of contamination across locations and species [63,64,65]. According to the estimated data resulting from CF and pollution indices (PLI and MPI), the degree of contamination in the present study by using liver, gills, and muscle of cultured and wild Barramundi fish can be classified as follows: gills > liver > muscle (Table 4).

### 3.3. Human Health Risk Assessment

#### 3.3.1. Estimated Daily Intake (EDI)

The estimated daily intake (EDI) was calculated using the oral reference dose (RfD) for a specific chemical [66], which characterizes daily exposure to harmful materials for avoiding any adverse effects on human health over lifetime [34]. The mean EDI value was higher in the cultured species in comparison to wild fish, which implied that the cultured fishes exert the highest exposure in both adult and children through the intake of the harmful elements. The EDIs of the metals Pb and Cr were higher than the RDA, while the EDIs of other metals were lower (Table 5). EDIs lower than the RDA suggested that the targeted groups of people might experience low or no health effects. However, determining a “acceptable limit” and a “unacceptable limit” based on doses less than the RDA/Rfd is not a stable measurement technique [34,38]. However, the results were far below the recommended threshold limit (Table 5).

#### 3.3.2. Non-Carcinogenic Health Risks (THQ, HI)

Individual hazard quotient (THQ) was calculated focusing on the metal’s uptake on a daily basis (EDI) (Table 6). According to the USEPA [33], the tolerable THQ threshold limit is 1. THQ values less than the unit limit imply that exposure to the pollutants would have no negative consequences for lifetime consumption [68]. In cultured Barramundi, the individual THQs of Pb, Cd, Cr, As, Mn, Cu, Zn were 0.001, 0.00002, 0.00013, 3.33 × 10^−5^, 1.43 × 10^−6^, 1 × 10^−5^, 0.00001, respectively, and were within the threshold value (Table 6). However, in wild Barramundi, the individual THQs of all the examined metals were also less than 1. So, the individual THQ of seven metals reveals no adverse health effects to humans. The findings emphasized the evaluation of hazard index (HI), in which exceeding HI unit (>1) expositions revealed an urgent issue of health risks for local consumers [69,70]. However, the HI results followed the THQ pattern and the investigated HI did not surpass the suggested limit, interpreting that the consumers (adult and children) would not experience any non-carcinogenic health effects through the consumption of the cultured and wild Barramundi fish species.

#### 3.3.3. Carcinogenic Health Risk (CR)

Carcinogenic risk (CR) was only calculated for Pb, Cd, Cr and As due to the availability of the carcinogenic potency slope factor of the carcinogens for those metals (Table 6) [69]. The estimated CR values for Pb, Cd, Cr and As were 8.22 × 10^−9^, 1.1 × 10^−7^, 2.07 × 10^−7^ and 1.31 × 10^−8^, respectively, in the cultured organisms and 8.96 × 10^−9^, 2.5 × 10^−8^, 1.66 × 10^−7^ and 2.38 × 10^−9^, respectively, in the wild species (Table 6). CR values lower than 10^−6^ show that the exposure of metals is negligible. In contrast, values above 10^−4^ indicate that the CR exposure is severe [31,71,72,73]. In our analysis, the CR values of cultured and wild Barramundi fish showed no carcinogenic risk as all the values were below 10^−6^.

### 3.4. Source Identification

Through the correlation analysis among the heavy metals [74], the origin and migration of metals can be detected, where the metals with a positive correlation may originate from the same sources [75]. To find out the source of the analyzed heavy metals, Pearson’s correlation analysis and principal component analysis (PCA) were performed (Table 7 and Table 8). We found that in the liver of cultured Barramundi, a strong positive correlation existed between Cr vs. Pb, and As vs. Pb, but no correlations were found between the metals in muscle and gill. However, in the muscle of wild Barramundi, a strong positive correlation was found between Pb and Cu, whereas in the liver of wild Barramundi, a positive correlation was found within Cr and Mn, Cr and Zn. In the gill tissue of wild Barramundi, a strong positive relationship exists between Cu and Mn.

In the PCA of heavy metals in the cultured Barramundi fishes, the first two components described about 87% of the total variance, whereas a total of seven components explained the total variance (Table 8). Component 1 was dominated by Cr (−0.62) and Cu (0.67), whereas Cr (0.64) and Mn (0.53) dominated component 2, indicating their close association and origination from same sources. The high loadings of Cd (0.90) were observed in component 3. Moreover, high loadings of Mn (−0.70) and Cu (0.52) were found in component 4 and As (0.56) and Zn (0.56) in component 5. Similar to cultured Barramundi, the first two components describe ~87% of the total variance the PCA of wild Barramundi samples, and seven components explained the total variance. Here, component 1 was dominated by Cr (0.95), and component 2 was dominated by Cu (0.44) and Mn (0.49), meaning the same sources of origin. High loadings of Cu (0.53) and As (−0.63) were observed in component 3.

However, in the cultured Barramundi, most of the metals were associated with the essential trace elements (Zn, Cu, Mn). Most of the essential elements might have been introduced from a common source of fish feed and feed residues in the culture system. So, there might be some non-essential elements which also co-existed with the essential elements in the fish food. On the contrary, some trace metals showed negative association with the essential elements, and those might have been introduced from other sources through waste mismanagement and disposal. As could be sourced from the underground water used in the culture process [74]. In the wild Barramundi, most of the metals (such as Cd, Pb and Cr) might have been deposited from the anthropogenic sources through sewage discharge, agricultural runoff, and industrial wastes. Some non-essential elements (i.e., Pb) were higher in wild fishes, possibly due to their longer life span resulting in longer exposure, and lower essential elements could be because they were not exposed to commercial feed [76].

## 4. Conclusions

The studied heavy metal concentrations in the Barramundi fish (Pb, Cd, Cr, As, Mn, Cu and Zn) were not significantly different between the cultured and wild specimens and their organs. The cultured fish were found to accumulate higher metals compared to wild ones. Comparing the organs of the Barramundi, the liver samples had the highest quantities of heavy metals. This indicates that the fish liver is the primary storage location for heavy metals in the fish body. Almost all the fish samples had the highest levels of Zn, Pb, and Cu in their livers and the highest levels of Pb, Cr, Cu, and Mn in their gills. Most of the examined heavy metals in the edible section of the fish were lower than the maximum permissible limits, except Pb and Cd in liver. Furthermore, the heavy metal concentrations in the edible sections of the fish were found to be safe for human consumption as the THQ values indicated no risk and the CR values of cultured and wild Barramundi fish showed no carcinogenic risk. The majority of these metals came from anthropogenic and geological sources. As a result, the potential health concerns posed by eating fish with high metal concentrations should be considered, as should the installation of regulatory measures to control the discharge of heavy metals. Heavy metal concentrations in the aquatic environment may influence cultured fish more than the wild fish, because the artificial food may have higher concentrations of heavy metals in them. Therefore, further investigation of heavy metals focusing on aquaculture water and sediment, river water and sediment, and natural and artificial feeding is recommended.

## Figures and Tables

**Table 1:** Heavy metal concentrations (mg/kg wet wt) in the liver, gills and muscle of cultured and wild Barramundi fish.

		Cultured			Wild	
**Liver**	**Max**	**Min**	**Mean ± SD**	**Max**	**Min**	**Mean ± SD**
Metals						
Pb	4.70	1.54	3.40 ± 1.25 ^a^	7.71	3.50	5.5 ± 1.69 ^b^
Cd	0.11	0.03	0.10 ± 0.04	0.04	0.01	0.02 ± 0.02
Cr	0.12	0.10	0.10 ± 0.03 ^a^	0.57	0.01	0.22 ± 0.21 ^a^
As	0.03	0.01	**0.03 ± 0.01** ^a^	0.02	0.01	**0.01 ± 0.01** ^b^
Mn	1.84	0.32	0.90 ± 0.7 ^a^	3.34	0.86	2.20 ± 0.91 ^b^
Cu	29.96	3.80	13.23 ± 10.5 ^a^	7.50	2.20	4.50 ± 1.98 ^b^
Zn	19.40	12.50	**15.28 ± 2.64** ^a^	19.40	10.95	**15.80 ± 1.9** ^a^
Gill						
Pb	5.21	1.84	2.80 ± 1.42 ^a^	3.82	2.32	3.00 ± 0.60 ^a^
Cd	0.05	0.02	0.04 ± 0.01 ^a^	0.02	0.03	**0.01 ± 0.01** ^a^
Cr	9.23	0.80	4.26 ± 4.023 ^a^	2.27	0.26	1.23 ± 0.76 ^b^
As	0.03	0.02	**0.02 ± 0.03** ^a^	0.10	0.02	0.03 ± 0.02 ^a^
Mn	3.80	0.94	1.94 ± 1.09 ^a^	5.33	0.24	2.90 ± 1.81 ^a^
Cu	5.50	0.37	2.50 ± 1.90 ^a^	2.40	0.13	1.40 ± 0.84 ^a^
Zn	24.80	11.60	**20.22 ± 5.13** ^a^	24.61	18.36	**20.62 ± 2.37** ^a^
Muscle						
Pb	1.89	1.26	1.22 ± 0.25 ^a^	1.73	0.98	1.33 ± 0.30 ^a^
Cd	0.03	0.07	0.02 ± 0.01 ^a^	0.01	0.02	0.05 ± 0.03 ^a^
Cr	1.61	0.10	0.52 ± 0.63 ^a^	0.80	0.03	0.42 ± 0.32 ^a^
As	0.02	0.01	**0.01 ± 0.04** ^a^	0.03	0.02	**0.02 ± 0.05** ^b^
Mn	0.44	0.12	0.26 ± 0.15 ^a^	0.72	0.18	0.43 ± 0.3 ^b^
Cu	0.65	0.42	0.54 ± 0.10 ^a^	0.65	0.330	0.50 ± 0.12 ^a^
Zn	4.90	2.43	**3.40 ± 1.0** ^a^	2.91	1.88	**2.40 ± 0.40** ^a^

The highest and lowest concentration in the respective organs are indicated as bold. Same letters (a,a) indicate no significant variation and different letters (a,b) significant variation at 5% signifcant levels.

**Table 2:** Maximum permissible limit (MPL) of heavy metals in fish muscles (mg/g wet wt.) according to international standards.

Competent Organization			Metal Concentration (mg/kg wet wt.)					References
	Pb	Cd	Cr	As	Mn	Cu	Zn	
Cultured Wild	1.22	0.022	0.522	0.011	0.262	0.543	3.35	Present Study
	1.33	0.005	0.42	0.002	0.429	0.502	2.40	
FAO (1983)	0.5	0.05	-	1	-	30	30	[51]
WHO (1989)	2	1	-	-	-	30	40	[52]
WHO (1985)	2	-	0.15	0.01	0.5	3	10–75	[56]
MAFF (2000)	2	0.2	-	-	-	20	50	[53]
EU (2008)	-	0.05–0.1	0.5	-	-	0.5–1	30	[61]
FEPA (2003)	2	-	0.15	-	0.5	1.3	75	[59]
Bangladesh Fish	0.30	0.25	1	5		5		[57]

FAO—Food and Agriculture; Organization of the United Nations; WHO—World Health Organization; EU—European Union; FEPA—Federal Environmental Protection Agency; MAFF—Ministry of Agriculture, Forestry and Fisheries.

**Table 3:** Concentration of the metals (mg/kg wet weight) in the fish sample muscles of cultured and wild Barramundi in different global studies.

River, Location	Nature of Species	Tissues	Pb	Cd	Cr	As	Mn	Cu	Zn	References
Noakhali, Bangladesh	Cultured	Muscle	1.22	0.022	0.522	0.011	0.262	0.543	3.35	Present Study
	Wild	Liver	1.33	0.005	0.42	0.002	0.429	0.502	2.40	
Honghu Lake, China	Cultured	Muscle	0.21	0	0.284	0	N/A	0.85	18.94	[23]
	Wild	liver	0.053	0.006	1.95	0	N/A	0.42	16.30	
Bangladesh	Cultured		0.090	0.003	1.274	1.486	2.512	1.138	1.850	[54]
Bangladesh	*Cultured*		-	0.004	0.590	0.042	-	0.874	16.205	[62]
	*T. nilotica*									
Bangladesh	Cultured	Muscle	-	0.006	0.577	0.045	-	1.035	20.324	[62]
	P. pangasius	liver								
Bangladesh	*Cultured*	Muscle	-	0.004	0.623	0.035	-	0.953	2.270	[62]
	*L. rohita*	liver								
Kasumigaura, Japan	Cultured		0.032	0.0074	0.076	0.18	0.177	0.332	5.45	[21]
	Wild		0.030	0.01	0.067	0.095	0.31	0.25	5.43	
Aegean Sea	Cultured	Muscle	1.03	0.27	0.17	N/A	7.25	3.87	45.1	[22]
	Wild	liver	0.84	0.17	0.15	N/A	6.53	2.96	43.6	
Iran	Cultured	Muscle	1.11	0.097	0.57	0.934	6.262	21.813	20.973	[50]
	Wild	liver	1.20	0.13	0.63	0.179	13.932	8.398	46.742	

**Table 4:** Values of the contamination factor (CF), pollution load index (PLI), and metal pollution index (MPI) of cultured and wild Barramundi fish.

Metals		Cultured			Wild	
Liver	CF	PLI	MPI	CF	PLI	MPI
Pb	0.17			0.25		
Cd	0.20			0.062		
Cr	0.001			0.002		
As	0.002	0.022	0.692	0.001	0.021	0.640
Mn	0.001			0.003		
Cu	0.294			0.099		
Zn	0.161			0.17		
Gill						
Pb	0.14			0.150		
Cd	0.113			0.023		
Cr	0.05			0.014		
As	0.002	0.032	0.970	0.002	0.021	0.681
Mn	0.002			0.003		
Cu	0.055			0.031		
Zn	0.213			0.217		
Muscle						
Pb	0.1			0.07		
Cd	0.072			0.02		
Cr	0.006			0.005		
As	0.001	0.012	0.26	0.0002	0.006	0.162
Mn	0.003			0.0005		
Cu	0.012			0.011		
Zn	0.04			0.03		

**Table 5:** A comparison between recommended daily allowance (RDA) and estimated daily intake (EDI).

Nature of Species	Elements	Mean Concentration (mg/kg)	Recommended Daily Allowance (mg/kg/ Person) [67]	EDIs (mg/day/ Person)
				Adult/Child
	Pb	1.22	0.25	0.0010/0.0043
	Cd	0.022	0.07	0.00002/0.0001
	Cr	0.522	0.23	0.0004/0.002
Cultured	As	0.011	0.15	0.00001/0.00004
	Mn	0.262	5	0.0002/0.001
	Cu	0.543	35	0.0004/0.002
	Zn	3.4		0.003/0.012
				Adult/Child
	Pb	1.33	0.25	0.0011/0.0047
	Cd	0.005	0.07	0.000004/0.00002
Wild	Cr	0.42	0.23	0.0003/0.001
	As	0.002	0.15	0.000002/0.00001
	Mn	0.43	5	0.0003/0.002
	Cu	0.50	35	0.0004/0.002
	Zn	2.40		0.0019/0.008

**Table 6:** Non-carcinogenic (THQ) and carcinogenic risk (CR) of metals of the cultured and wild species.

Metals		Cultured			Wild	
	THQ	HI	CR	THQ	HI	CR
Pb	0.001		8.22 × 10^−5^	5.3 × 10^−4^		8.96 × 10^−9^
Cd	2 × 10^−5^		1.1 × 10^−7^	3.96 × 10^−6^		2.5 × 10^−8^
Cr	1. × 10^−3^		2.07 × 10^−7^	1.1 × 10^−4^		1.67 × 10^−7^
As	3.33 × 10^−5^	7.07 × 10^−4^	1.31 × 10^−8^	5.29 × 10^−6^	6.66 × 10^−4^	2.38 × 10^−9^
Mn	1.43 × 10^−6^		0	2.44 × 10^−6^		
Cu	1 × 10^−5^		0	9.9 × 10^−6^		
Zn	1 × 10^−5^		0	6.34 × 10^−6^		

**Table 7:** Correlation matrix of the metal concentrations in muscle, liver and gill tissues of cultured and wild Barramundi fish.

	Pb	Cd	Cr	As	Mn	Cu	Zn
Cultured Barramundi							
Muscle							
Pb	1						
Cd	0.21	1					
Cr	−0.41	−0.17	−0.20				
As	−0.06	−0.02	0.55	1			
Mn	−0.41	0.31	−0.29	0.56	1		
Cu	0.17	−0.87	−0.29	0.15	−0.55	1	
Zn	0.29	−0.62	−0.20	0.64	−0.11	0.82	1
Liver							
Pb	1						
Cd	0.82	1					
Cr	0.89 *	0.61	1				
As	1.00 *	0.83	0.87	1			
Mn	0.75	0.56	0.48	0.79	1		
Cu	0.65	0.86	0.65	0.67	0.26	1	
Zn	0.60	0.50	0.53	0.66	0.76	0.54	1
Gill							
Pb	1						
Cd	−0.02	1					
Cr	−0.70	0.06	1				
As	−0.43	−0.32	−0.30	1			
Mn	0.07	0.86	−0.30	−0.03	1		
Cu	0.76	0.49	−0.77	−0.16	0.57	1	
Zn	0.33	0.59	−0.49	0.10	0.50	0.82	1
Wild Barramundi							
Muscle							
Pb	1						
Cd	−0.33	1					
Cr	0.68	0.39	1				
As	−0.32	0.75	0.42	1			
Mn	0.87	−0.45	0.50	−0.22	1		
Cu	0.93 *	0.01	0.78	−0.18	0.74	1	
Zn	0.56	−0.07	0.28	−0.18	0.74	0.65	1
Liver							
Pb	1						
Cd	−0.29	1					
Cr	−0.23	0.70	1				
As	0.37	−0.57	−0.35	1			
Mn	−0.25	0.52	0.97 *	−0.29	1		
Cu	0.36	0.52	−0.05	−0.52	−0.25	1	
Zn	−0.39	0.87	0.92 *	−0.66	0.84	0.21	1
Gill							
Pb	1						
Cd	0.18	1					
Cr	0.55	0.02	1				
As	−0.44	−0.27	−0.14	1			
Mn	0.66	0.38	0.85	0.02	1		
Cu	0.86	0.36	0.81	−0.21	0.95 *	1	
Zn	−0.52	−0.22	−0.91 *	−0.15	−0.97 *	−0.87	1

*p* < 0.05 is significant and *p* < 0.01 * is mostly significant. Significant values are indicated in bold.

**Table 8:** PCA results for heavy metals in the cultured and wild Barramundi fishes.

	PC 1	PC 2	PC 3	PC 4	PC 5	PC 6	PC 7
1	−2.3475	−0.8181	−0.2565	−0.17335	−1.3231	−0.69377	−0.4862
2	0.2175	−1.5861	0.6601	1.1443	0.49778	2.0035	−0.62232
3	0.11952	−1.1878	0.78617	0.6952	−0.33164	0.22519	−0.13874
4	−0.12468	−1.2071	0.53131	−1.6902	0.86054	−0.93909	0.57079
5	0.21705	−1.2411	0.37336	−0.94002	−0.29796	0.081791	0.88353
6	−2.3104	1.4381	−0.33613	−0.3772	0.60962	0.87572	−0.16499
7	0.50843	0.52725	0.39175	0.033328	−0.90721	−1.2461	0.58149
8	0.026057	0.99566	0.314	0.58778	0.30979	1.2507	1.5251
9	0.20131	0.89526	0.83414	−0.11634	1.4347	−0.3482	1.4285
10	0.15533	0.9525	1.6299	1.2301	0.11953	−1.0061	−1.8325
11	0.082446	−0.59466	−2.6017	1.7433	1.0272	−0.93055	0.37858
12	0.89262	0.64168	−1.077	−1.012	−1.5426	1.4946	−0.32344
13	0.93056	0.13045	−0.70893	−0.59126	−0.07717	−0.54195	−0.6027
14	0.7807	0.41904	−0.55634	−1.2326	1.257	0.14176	−1.7887
15	0.65111	0.63482	0.015851	0.69903	−1.6366	−0.36747	0.5916

## Data Availability

Data are provided in the article.

## Supplementary Materials

The following are available online at https://www.mdpi.com/article/10.3390/toxics10080410/s1, Table S1: Spectral lines used in emission measurements and the instrumental detection limit for the elements measured by using AAS.
