# Supplementary material for: Heavy Metals Accumulation in Tissues of Wild and Farmed Barramundi from the Northern Bay of Bengal Coast, and Its Estimated Human Health Risks
Source: Toxics. 2022 Jul 22;10(8):410. doi: 10.3390/toxics10080410 (PMC9330387; doi:10.3390/toxics10080410)
Supplement: Supplementary file 1 [file toxics-10-00410-s001.zip › toxics-1815723-supplementary.pdf]

toxics MDPI

Supplementary Materials: Heavy Metals Accumulation in Tissues of Wild and Farmed Barramundi from the Northern Bay of Bengal Coast, and Its Estimated Human Health Risks

Tanha Tahity, Md. Rakeb Ul Islam, Nurer Zaman Bhuiyan, Tasrina Rabia Choudhury, Jimmy Yu, Md. Abu Noman, Mohammad Mozammal Hosen, Shamshad B. Quraishi, Bilal Ahamad Paray, Takaomi Arai and Mohammad Belal Hossain

Table S1. Spectral lines used in emission measurements and the instrumental detection limit for the elements measured by using AAS.

Elements	Wavelengths (nm)	Instrumental detection limit (mg/L)
Hg	253.7	
As	193.7	
Pb	217.0	0.013
Cr	357.9	0.0054
Cd	228.8	0.0028
Mn	279.5	0.0016
Cu	324.8	0.004
Zn	213.9	0.0033
